# Thermal Stability and Electrical Properties of High-Pressure-Molded Nanocomposites Containing Fast Ion-Conductive δ-Bi_2_O_3_ Phase

**DOI:** 10.3390/nano16120753

**Published:** 2026-06-16

**Authors:** Aleksander Szpakiewicz-Szatan, Jerzy E. Garbarczyk, Sylwester J. Rzoska, Tomasz K. Pietrzak, Jan Mizeracki

**Affiliations:** 1 Institute of High Pressure Physics Polish Academy of Sciences (Unipress), Sokołowska 29/37, 01-142 Warsaw, Poland; janekm@unipress.waw.pl; 2 Faculty of Physics, Warsaw University of Technology, Koszykowa 75, 00-662 Warsaw, Poland; jerzy.garbarczyk@pw.edu.pl (J.E.G.); tomasz.pietrzak@pw.edu.pl (T.K.P.)

**Keywords:** δ-Bi_2_O_3_, high-pressure methods, nanocrystallization, nanocomposites, oxygen conductors, polymorphism of Bi_2_O_3_

## Abstract

The report presents the electrical, structural, and microstructural properties of high-pressure–high-temperature-treated (HPHT) composites composed of δ-like Bi_2_O_3_ nanograins embedded in an aluminosilicate glassy matrix. Nanocomposites were obtained by heat treatment of the Bi_2_O_3_-Al_2_O_3_-SiO_2_ ternary glass system, followed by high-pressure molding (above 750 MPa). The total oxygen conductivity σ_t_ of the studied nanocomposites was high and approached a value of 4.5 × 10^−4^ S/cm at 600 °C. Due to HPHT treatment, we could also determine the intragrain conductivity of δ-Bi_2_O_3_ nanocrystallites. In this case, the value of σ_δ_ was even higher and was equal to 1.3 × 10^−3^ S/cm at 600 °C. It was also possible to study the temperature dependence of intragrain conductivity, showing two activation energies, which probably reflect the order–disorder transition within the sublattice of mobile O^2−^ ions. The obtained nanocomposites exhibited promising properties for applications in electrochemical devices operating in the intermediate temperature range from 300 to 600 °C.

## 1. Introduction

Bismuth sesquioxide, Bi_2_O_3_, is a compound of rich polymorphism [1,2,3,4,5] and can exhibit either semiconductor [6], ferroelectric [7,8], or fast oxygen-ion conductor properties [9], depending on the crystalline phase: α, β, γ, and δ.

This promising material is considered for use in a wide range of applications such as supercapacitors, photonic sensors, capsules in nuclear energetics, and solid oxide fuel cells [8,9,10,11,12,13,14]. Due to the extremely high conductivity of O^2−^ ions, the face-centered cubic fluorite phase (δ-Bi_2_O_3_) is an attractive material for applications in electrochemical devices such as oxygen sensors or as an electrolyte in fuel cells [15,16,17,18,19,20,21,22]. A major disadvantage of the δ phase is that it is stable only over a narrow, high-temperature (1000–1100 K) range [1,23,24,25,26].

In our laboratory, we avoided this drawback by preparing Bi_2_O_3_-SiO_2_-Al_2_O_3_ glasses, which we then subjected to thermal nanocrystallization [27,28,29,30,31,32]. Such composites contain nanograins of δ-Bi_2_O_3_ confined in a glassy matrix, which are stable down to room temperature and even lower [15,28,29,30,31,32]. Preparation of pure Bi_2_O_3_ glass is practically impossible, and therefore, the addition of glass formers like SiO_2_ and Al_2_O_3_ is required. This can be achieved by intentional addition [32] or even more simply, by melting α-Bi_2_O_3_ in a ceramic crucible containing glass-forming silica and alumina [15,30,31]. Above the melting temperature of α-Bi_2_O_3_, both oxides diffuse from the crucible shell to the melt, making glass formation possible.

High-pressure high-temperature (HPHT) treatment of glasses may permanently alter their structural, thermal, and electrical properties [33,34,35,36,37,38]. In oxide glasses, densification exceeding 10%, an extraordinary Young’s modulus, and enhanced surface hardness were observed [36,37,38,39,40]. Those notable changes remain after decompression and are stable for years at ambient conditions [38,39,40,41,42,43]. However, one may suspect that the extraordinary properties of HPHT-treated materials could not remain stable at elevated temperatures under ambient pressure.

In our previous research, HPHT treatment of alkali metal phospho-olivines (such as LiFe_0.75_V_0.10_PO_4_ [41,42] and NaFePO_4_ [43,44]) has shown that HPHT treatment of relatively large (*m* = 5–10 g) samples at high (but still reasonably moderate) pressure of *P*~1 GPa allowed for the growth of nanocrystalline phases different from those obtained at ambient pressure. In the case of the mentioned phospho-olivines, this led to an increase in DC conductivity by over two orders of magnitude at room temperature and ambient pressure. The structure of those samples remained stable for at least a year [45].

Authors’ previous research [15] has shown that depending on the pressure applied during the HPHT treatment, Bi_2_O_3_-based glass can transform into other phases: body-centered cubic γ-Bi_2_O_3_ [29,30], face-centered cubic δ-Bi_2_O_3_ [46], or tetragonal β-Bi_2_O_3_ phase [47,48]. Depending on conditions (not fulfilled in this study), it could introduce some silicon oxide from the glassy matrix into crystallites and create potentially ferroelectric (either orthorhombic or close-to-90° monoclinic) bismuth silicate Bi_2_O_5_Si [7,32,49]. Results achieved in our studies described in [15] allowed us to propose a *P*-*T* map of phase transitions for the Bi_2_O_3_-Al_2_O_3_-SiO_2_ ternary system.

The aim of this work is to study the electrical properties of Bi_2_O_3_-Al_2_O_3_-SiO_2_ composites prepared by us that contain nanograins of superionic δ-Bi_2_O_3_ phase. This topic was also studied in our earlier papers [28,29,30,31,32], but the novelty of the present work is the use of high pressures in order to obtain samples with better properties. Our main focus was to study the impact of HPHT annealing on the ionic conductivity of this composite. While determining the total ionic conductivity of glass ceramics is quite standard, determining the intragrain conductivity of the δ-Bi_2_O_3_ phase is more challenging.

## 2. Materials and Methods

In order to prepare samples, monoclinic α-Bi_2_O_3_ [15,50,51] (Acros Organics, Geel, Belgium, Bismuth (III) oxide, 99.9% extra pure) was pulverized in a zirconia mortar and placed in a ceramic Al_2_O_3_ crucible. The crucible’s glaze contained SiO_2_ and Al_2_O_3_ oxides (VWR, Radnor, PA, USA, 459–0211 DIN). The powder was then heated to 1373 K and held at this temperature for 30 min, followed by melt quenching [15,28,29,30,31,32,52]. The quenching consisted of pouring the melt onto a steel plate (kept at RT) and covering the liquid sample with a second similar plate. This resulted in a glassy material, which was confirmed by XRD analysis. All structure studies (amorphous and nanocrystalline samples) were carried out at ambient conditions utilizing a PANalytical Empyrean X-ray diffractometer with CuK_α_ λ = 1.54 Å (PANalytical B.V., Almelo, The Netherlands). The data were analyzed using PANalytical High Score Plus software (Ver. 4.7.0.24755, PANalytical B.V., Almelo, The Netherlands) with the JCPDS-ICDD (The International Centre for Diffraction Data, Newtown Square, PA, USA) database.

The differential thermal analysis (DTA) of the obtained glass is part of our previous publication on this topic [15].

**Figure 1:**
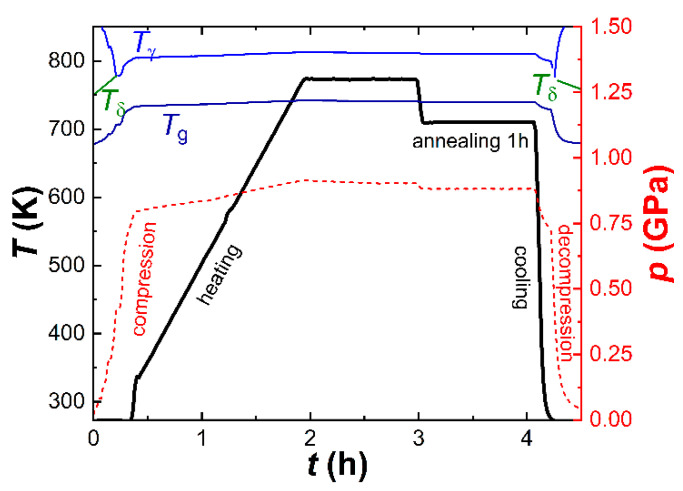
The HPHT treatment of the studied samples. Solid black line—temperature, dashed red line—pressure. *T_g_*—glass transition temperature, *T_δ_*—crystallization temperature of δ-Bi_2_O_3_, *T_γ_*—crystallization temperature of γ-Bi_2_O_3_. The pressure-dependent glass-transition and crystallization temperatures are based on the authors’ previous *P*-*T* diagram of phase transitions [15].

To prepare samples studied in this work, we subjected them to high-pressure high-temperature (HPHT) treatment, developed at our laboratory [15,42,43,44,45,46] and described in Figure 1. The scenario for this treatment was proposed based on our previously published *P*-*T* diagram of phase transitions [15]. In the HPHT experiment, Bi_2_O_3_-based glass (Bi_2_O_3_-Al_2_O_3_-SiO_2_ ternary system) was first thermally nanocrystallized into δ-Bi_2_O_3_ [15,29,30,31,32]. Next, the nanocrystalline material was placed in a pressure chamber, compressed to circa 770 MPa, heated above the glass transition temperature, and maintained under these conditions for 1 h. Next, the material was cooled down slightly to below the glass transition temperature and annealed for 1 h. Finally, the material was cooled down to room temperature and decompressed to atmospheric pressure. The HPHT procedure is illustrated in Figure 1.

The microstructure of the samples was examined using a Zeiss Ultra Plus SEM. Glasses (Carl Zeiss AG, Oberkochen, Germany) and nanomaterials of the Bi_2_O_3_-Al_2_O_3_-SiO_2_ system were measured using the EDS method with the use of the EDS Bruker Quantax 400 (Bruker Corporation, Billerica, MA, USA) extension of the SEM equipment. Our EDS results show that the weight percentage of the nanocomposite sample before HPHT annealing was as follows: Bi (84.57 ± 5.95)%, O (9.81 ± 2.90)%, Si (3.42 ± 0.35)%, and Al (2.20 ± 0.27)%. The weight percentage in the HPHT-annealed sample remained very close at Bi (81.64 ± 5.74)%, O (10.72 ± 3.17)%, Si (4.63 ± 0.47)%, and Al (3.0 ± 0.36)%. Atomic compositions were: Bi (33.15 ± 2.33)%, O (50.21 ± 14.86)%, Si (9.97 ± 1.01)% and Al (6.68 ± 0.82)% and Bi (29.22 ± 2.06)%, O (50.11 ± 14.83)%, Si (12.34 ± 1.25)% and Al (8.33 ± 1.02)%, respectively.

Electrical properties were studied using impedance spectroscopy (IS) [29,30,52,53] on polished samples with sputtered Pt electrodes. Conductivity values were determined from the measured resistances of the samples and their geometric factors. Measurements were performed using a Novocontrol Alpha-A spectrometer (Novocontrol Technologies GmbH & Co. KG, Hundsangen, Germany) in a temperature range from RT to 900 K and at frequencies between 10 mHz and 10 MHz. Data acquisition was controlled by the KURA software (version 0.7) developed in our laboratory [53].

## 3. Results

**Figure 2:**
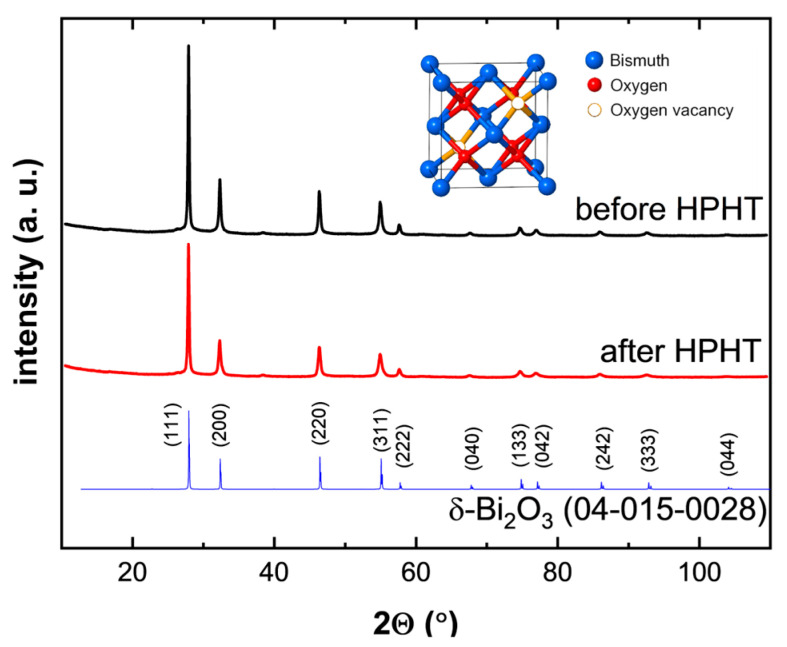
XRD patterns of the studied composites before and after HPHT treatment. The only crystalline phase is δ-Bi_2_O_3_ (JCPDS ICDD 04-15-0028). Silica and alumina form a glass matrix of the composites.

Figure 2 shows the fluorite-like unit cell of δ-Bi_2_O_3_ and X-Ray diffraction patterns of the studied composites before and after HPHT treatment. The HPHT treatment did not change the crystalline phase of δ-Bi_2_O_3_ nanograins, nor did it form any other crystalline phases in the Al_2_O_3_-SiO_2_ glassy matrix. The positions of the four most significant peaks and their relative proportions did not change (only a slight shift of 0.03–0.05° was observed). The crystallite sizes were determined by the Scherrer [54] formula on the basis of peak positions and their shapes. The crystallite sizes calculated for the four most significant peaks were 95 nm, 66 nm, 48 nm, and 36 nm before, and 100 nm, 66 nm, 48 nm, and 34 nm after HPHT. The average crystallite sizes were 61 (45) nm and 62 (50) nm, respectively; the difference between the pairs was within the limits of experimental error.

**Figure 3:**
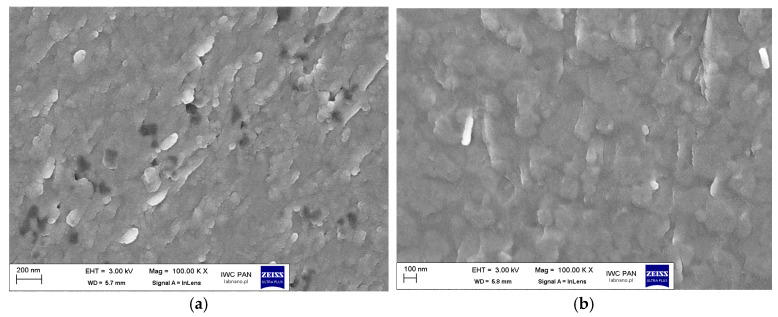
SEM images of studied composites: (**a**) before and (**b**) after HPHT treatment.

Figure 3a,b shows that HPHT treatment successfully altered the microstructure of the samples, making them more compact. The distance between larger nanograins of δ-Bi_2_O_3_ seems to be reduced. It is also worth noting that the average size of nanograins seen in Figure 3b (c.a. 100 nm) is slightly larger than the average size of nanocrystallites determined from XRD. This is understandable because a grain, visible in SEM, consists of a crystalline core and a disordered shell.

**Figure 4:**
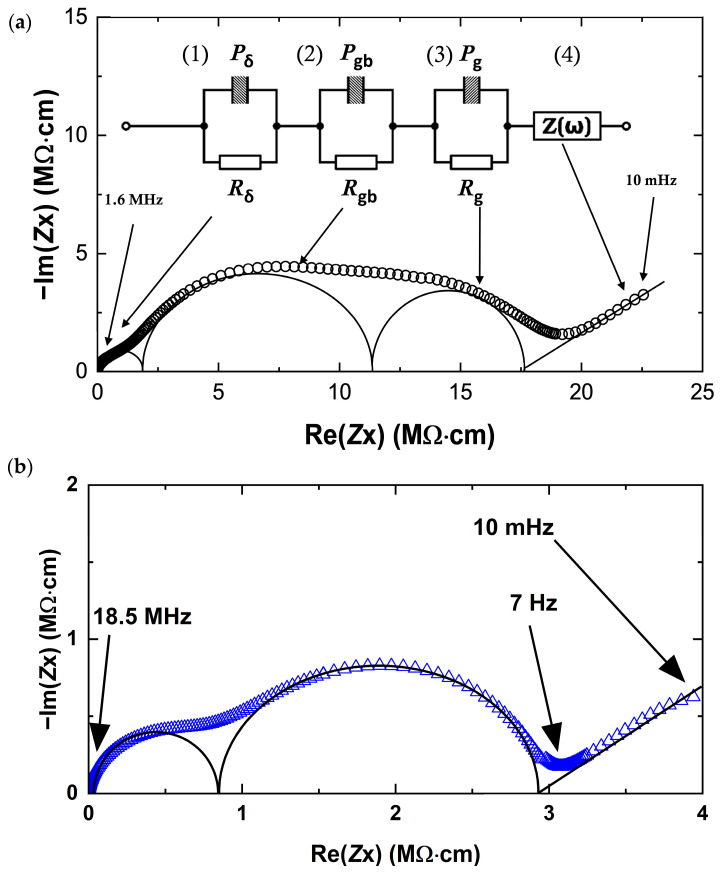
(**a**) Impedance diagram of HPHT-treated composite containing nanograins of δ-Bi_2_O_3_ (*T* = 537 K, during cooling). The electric equivalent circuit consists of four elements described in the text related to ionic polarization of (1) grains, (2) grain boundaries and (3) glass with additional (4) impedance related to diffusion through porous Pt electrode. Black circles are measurement data, lines are responses of parts of equivalent circuit. (**b**) Impedance diagram of thermally nanocrystallized glass containing nanograins of δ-Bi_2_O_3_, not detected by impedance spectroscopy (*T* = 539 K, during cooling). Blue triangles are measurement data, lines are responses of parts of equivalent circuit. Measurement frequencies were marked with arrows. Impedance data were normalized with the samples’ geometric parameters. Thickness and surface area of samples used in measurements before and after HPHT treatment (respectively): 0.89 mm, 11 mm^2^, and 0.56 mm, 54.94 mm^2^.

Electrical properties were studied using impedance spectroscopy across a wide range of frequencies. Considering the SEM images (Figure 3b), we proposed an equivalent electric circuit shown in Figure 4 together with a typical impedance diagram. The circuit consists of four elements corresponding to: (1) ionic polarization of δ-Bi_2_O_3_ nanograins, (2) ionic polarization of grain boundaries, (3) ionic polarization of thin glassy layers between grains, and (4) impedance *Z*(ω) related to diffusion of O^2−^ ions in a porous Pt electrode [55,56].

*R_δ_*, *R_gb_*, and *R_g_* denote the resistances of nanograins, grain boundaries, and glass areas, respectively. Capacitances corresponding to the polarization effects mentioned above are marked with *P_δ_*, *P_gb_*, and *P_g_* (more precisely, these are *constant phase elements* commonly used in impedance spectroscopy [57,58,59]). Low-frequency impedance *Z*(ω) is not the focus of this study, because it concerns the diffusion of O^2−^ ions in the porous electrode area. Most interesting for us, the electrical conductivity of oxygen ions in nanograins (intragrain conductivity) reveals itself at the highest frequencies (Figure 4). In the intermediate frequency range, transport phenomena occur at the grain boundaries [29,31,60,61] and in the glassy matrix area. The considerable sizes of the impedance arcs corresponding to those processes (Figure 4) indicate that the contribution of intergrain areas to the total resistance is also considerable. The total resistivity (conductivity σ_t_) of the studied samples was determined from the Re*Z_x_*—intercept. In Figure 4a, one can see experimental points and tentative fitted impedance arcs, corresponding to the polarization phenomena mentioned above. In our case, due to HPHT treatment, the separation of those processes and the determination of intragrain ionic conductivity σ_δ_ were possible without complex, time-consuming theoretical fitting [31]. On the other hand, the extraction of the intragrain contribution from the total impedance diagram is extremely difficult without HPHT treatment. In Figure 4b, related to thermally nanocrystallized glasses, the polarization of intragrains is not visible. In our earlier studies, we could detect this process using only ultra-high frequencies approaching 10 GHz [31]. However, applying such frequencies requires special instruments and a very careful electric shielding to avoid the influence of parasitic inductances.

If we compare the impedance diagrams in Figure 4a,b, we can see that the proportions between the impedances of residual glass and grain boundaries were reversed after HPHT treatment. The relative contribution of thin glassy layers to the total resistivity decreased, while the relative contribution of grain boundaries increased. Additionally, the contribution of intragrain resistivity was clearly visible. Total resistivity after HPHT treatment, quite unexpectedly, slightly increased (Figure 4a,b). This is probably due to closer contacts between grains and the build-up of a ramified network of grain boundaries. This minor disadvantage is, however, compensated by the remarkable possibility to study small grains of δ-Bi_2_O_3_.

**Figure 5:**
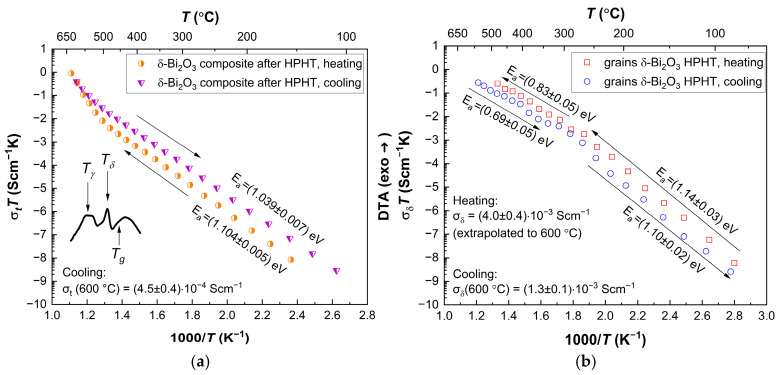
Temperature dependencies of ionic conductivity of HPHT-treated composite during heating and cooling: (**a**) total ionic conductivity, (**b**) intra-grain ionic conductivity. The inset in Figure 5a shows a DTA thermogram indicating characteristic temperatures of thermally crystallized glass [15].

Based on impedance diagrams recorded at various temperatures, during heating and cooling, the total conductivity of the HPHT-treated nanocomposite was determined and presented in Arrhenius coordinates in Figure 5a. Since the composite still contains a residual glassy matrix, we inserted in this figure the DTA thermogram obtained for the initial glass [15]. Although the composition of the glassy matrix may differ from that of the initial glass, the attached thermogram may facilitate the analysis of the Arrhenius plots. It can be observed that at about 450 °C, the ionic conductivity sharply increases, apparently due to the glass transition and redundant crystallization of the δ phase. The upper limit of conductivity measurements was determined by the phase transition from δ to the less conductive and metastable γ phase.

The total oxygen conductivity was equal to (4.5 ± 0.4) × 10^−4^ S/cm at 600 °C, which is a promising value for applications in intermediate-temperature electrochemical devices based on solid oxide electrolytes. High activation energies (≈1 eV), describing effective ionic conduction, are typical for oxygen conductors [62] because the radius of the O^2−^ anion is relatively large and equals 1.4 Å [62,63].

Figure 5b shows Arrhenius plots related to the intragrain conductivity of the δ-phase extracted from impedance diagrams like that in Figure 4a. In this case, we observe a change in activation energy from higher values (about 1 eV) below 300 °C to considerably lower ones (0.7–0.8 eV) above that temperature. Details concerning the conductivity runs are discussed below. It is worth noting that the intragrain conductivity of the δ phase reaches a high value of (1.3 ± 0.1) × 10^−3^ S/cm at 600 °C during cooling.

## 4. Discussion

Area-specific resistance required for practical fuel cell operation should not exceed 0.1 Ωcm^−2^ [64]. A 1-mm-thick layer requires a conductivity of at least 10^−1^ Scm^−1^; lower conductivity imposes a limit on layer thickness [64,65,66]. Materials such as (Y_2_O_3_)_0.08_(ZrO_2_)_0.92_, Ce_0.9_Gd_0.1_O_1.95_, La_0.9_Sr_0.1_Ga_0.9_Mg_0.1_O_3_, (BiO_1.5_)_0.88_(DyO_1.5_)_0.08_(WO_3_)_0.04_, and (Sc_2_O_3_)_0.08_(CeO_2_)_0.01_(ZrO_2_)_0.91_, could exhibit conductivities in the range of 10^−3^ to 10^−2^ S/cm at 500 °C [64,67,68,69,70,71]. Similarly, tungsten-stabilized Bi_3_Y_0.90_W_0.1_O_6.15_ could reach 3 × 10^−2^ S/cm at 600 °C [19], while thermally nanocrystallized δ-Bi_2_O_3_ reaches a total conductivity of 5 × 10^−5^ S/cm at 500 °C [31]. Our sample reached a total conductivity of (4.5 ± 0.4) × 10^−4^ S/cm at 600 °C, which is a promising starting point for further refinement before practical application, especially considering the lowered activation energy (1.039 ± 0.007 eV), when compared with that reported in previous studies (1.30 eV) [31].

Superionic δ-Bi_2_O_3_ = δ-BiO_1.5_ exhibits a fluorite (CaF_2_) structure, whose oxygen sublattice contains 25% vacancies [72]. Such conditions strongly facilitate fast ionic transport by the vacancy mechanism [72,73,74,75]. On the other hand, a substantial disorder in the O^2−^ sublattice cannot be stable at lower temperatures, where oxygen vacancies tend to form an ordered superstructure within the fluorite structure [76,77,78].

A change in the activation energy of ionic conductivity from a higher to a lower value is a characteristic feature of many superionic conductors, e.g., sodium β″-alumina or β-PbF_2_, which, like δ-Bi_2_O_3_, exhibits a fluorite structure [79,80,81,82,83,84,85,86,87,88]. In this class of fast ion conductors, a second-order phase transition occurring in the sublattice of mobile ions has been postulated. Fast ion conduction at high temperatures is the result of strong disorder of the “molten” sublattice of ions. In the lower-temperature range, a defective sublattice tends to order, which increases the activation energy. It is obvious that the degree of ordering depends on temperature and that this fundamental issue requires further studies. Transport of ions in an ordered sublattice is more difficult because the activation energy consists not only of *migration energy* but also of *defect creation energy* (creation of an oxygen vacancy in our case) [89].

In Figure 5b, one can see that the conductivity during cooling is unexpectedly slightly lower than during heating. We think that this may be caused by a stronger rate of vacancy ordering upon cooling. Impedance measurements, in situations where the sublattice of mobile ions evolves over time, are quite challenging. In our previous studies [29,31], we could not detect (in Arrhenius plot) the changing point of activation energy. This was caused by difficulties in determining intragrain conductivity. In this study, due to HPHT treatment of the composites, this determination was easier. The reduction in conductivity during cooling suggests that the HPHT-induced effect is further modified during thermal cycling. The intragrain activation energy (0.69 ± 0.05 eV), while higher than that reported by Takahashi (0.4–0.5 eV) at temperatures above 730 °C, is remarkably lower than that previously observed in thermally nanocrystallized δ-Bi_2_O_3_, which was not HPHT-annealed (0.96 eV) [31].

## 5. Conclusions

In this work, we applied high-pressure high-temperature treatment (HPHT) to obtain composites containing nanograins of the superionic δ-Bi_2_O_3_ phase. This treatment allowed us to determine the intragrain conductivity of O^2−^ ions over a wide temperature range. The electrical properties of the composites are very promising from an application standpoint. It was also possible to detect changes in activation energy that may indicate an order–disorder transition in the sublattice of mobile oxygen ions. This hypothesis, however, requires further research, for example, using neutron diffractometry.

## Data Availability

Data is available at public repository: 10.6084/m9.figshare.32358351.

## Abbreviations

The following abbreviations are used in this manuscript:

HPHT	High Pressure-High Temperature
XRD	X-Ray Diffraction
SEM	Scanning Electron Microscope
IS	Impedance Spectroscopy
EDS	Energy-Dispersive X-ray Spectroscopy

## References

[1] Levin E.M., Roth R.S. (1964). Polymorphism of bismuth sesquioxide. I. pure Bi_2_O_3_. J. Res. Natl. Bur. Stand. A Phys. Chem..

[2] Harwig H.A., Gerards A.G. (1979). The polymorphism of bismuth sesquioxide. Thermochim. Acta.

[3] Drache M., Roussel P., Wignacourt J.P. (2007). Structures and oxide mobility in Bi-Ln-O materials: Heritage of Bi_2_O_3_. Chem. Rev..

[4] Cornei N., Tancret N., Abraham F., Mentré O. (2006). New ε-Bi_2_O_3_ metastable polymorph. Inorg. Chem..

[5] Gualtieri A.F., Immovilli S., Prudenziati M. (1997). Powder X-ray diffraction data for the new polymorphic compound ω-Bi_2_O_3_. Powder Diffr..

[6] Utami B.A., Sutanto H., Hidayanto E. (2022). Recent advances in doped Bi_2_O_3_ and its photocatalytic activity: A review. Int. J. Res. Rev..

[7] Taniguchi H., Kuwabara A., Kim J., Kim Y., Moriwake H., Kim S., Hoshiyama T., Koyama T., Mori S., Takata M. (2013). Ferroelectricity driven by twisting of silicate tetrahedral chains. Angew. Chem. Int. Ed. Engl..

[8] Yang Q., Hu J., Fang Y.W., Jia Y., Yang R., Deng S., Lu Y., Dieguez O., Fan L., Zheng D. (2023). Ferroelectricity in layered bismuth oxide down to 1 nanometer. Science.

[9] Maeder T. (2013). Review of Bi_2_O_3_ based glasses for electronics and related applications. Int. Mater. Rev..

[10] Astuti Y., Mei R., Darmawan A., Arnelli J., Widiyandari H. (2022). Enhancement of electrical conductivity of bismuth oxide/activated carbon composite. Sci. Iran..

[11] Jeong I., Jeong S.J., Yun B.H., Lee J.W., Lee C.W., Jung W., Lee K.T. (2022). Physically driven enhancement of the stability of Bi_2_O_3_-based ionic conductors via grain boundary engineering. NPG Asia Mater..

[12] Singh S., Sahoo R.K., Shinde N.M., Yun J.M., Mane R.S., Chung W., Kim K.H. (2019). A symmetric faradaic assembly of Bi_2_O_3_ and MnO_2_ for a high-performance hybrid electrochemical energy storage device. RSC Adv..

[13] Luo W., Li F., Li Q., Wang X., Yang W., Zhou L., Mai L. (2018). Hetero-structured Bi_2_S_3_−Bi_2_O_3_ nanosheets with a built-in electric field for improved sodium storage. ACS Appl. Mater. Interfaces.

[14] Kilner J.A., Burriel M. (2014). Materials for intermediate-temperature solid-oxide fuel cells. Annu. Rev. Mater. Res..

[15] Szpakiewicz-Szatan A., Pietrzak T.K., Sierakowski K., Boćkowski M., Rzoska S.J., Garbarczyk J.E., Starzonek S. (2024). Nanocrystallization of Bi_2_O_3_ based system from the glassy state under high compression. Materialia.

[16] Wachsman E.D., Lee K.T. (2011). Lowering the temperature of solid oxide fuel cells. Science.

[17] Wachsman E.D., Marlowe C.A., Lee K.T. (2012). Role of solid oxide fuel cells in a balanced energy strategy. Energy Environ. Sci..

[18] Borowska-Centkowska A., Leszczynska M., Wrobel W., Malys M., Hull S., Krok F., Abrahams I. (2020). Stability of tungsten-doped δ-Bi_3_YO_6_. Solid State Ion..

[19] Borowska-Centkowska A., Leszczynska M., Wrobel W., Malys M., Krynski M., Hull S., Krok F., Abrahams I. (2017). Structure and conductivity in tungsten doped δ-Bi_3_YO_6_. Solid State Ion..

[20] Shao Z., Haile S.M. (2004). A high-performance cathode for the next generation of solid-oxide fuel cells. Nature.

[21] Harwig H.A., Gerards A.G. (1978). Electrical properties of the α, β, γ, and δ phases of bismuth sesquioxide. J. Solid State Chem..

[22] Basu S., Basu S. (2007). Future directions of fuel cell science and technology. Recent Trends in Fuel Cell Science and Technology.

[23] Schröder F., Bagdassarov N., Ritter F., Bayarjargal L. (2010). Temperature dependence of Bi_2_O_3_ structural parameters close to the α–δ phase transition. Phase Transit..

[24] Risold D., Hallstedt B., Gauckler L.J., Lukas H.L., Fries S.G. (1995). The bismuth-oxygen system. J. Phase Equilibria.

[25] Matsumoto A., Koyama Y., Tanaka I. (2010). Structures and energetics of Bi_2_O_3_ polymorphs in a defective fluorite family derived by systematic first-principles lattice dynamics calculations. Phys. Rev. B Condens. Matter Mater. Phys..

[26] Kilner J., Brook R. (1982). A study of oxygen ion conductivity in doped non-stoichiometric oxides. Solid State Ion..

[27] Garbarczyk J.E., Wasiucionek M., Jozwiak P., Nowinski J.L., Julien C.M. (2009). Novel nanomaterials based on electronic and mixed conductive glasses. Solid State Ion..

[28] Kruk-Fura P., Garbarczyk J.E. (2022). Studies on δ-Bi_2_O_3_ Based Stabilized at Room Temperature by Novel Methods. Appl. Sci..

[29] Pietrzak T.K., Wasiucionek M., Garbarczyk J.E. (2021). Towards higher electric conductivity and wider phase stability range via nanostructured glass-ceramics processing. Nanomaterials.

[30] Pietrzak T.K., Jarocka A., Jastrzebski C., Plocinski T., Wasiucionek M., Garbarczyk J.E. (2021). Facile and reproducible method of stabilizing δ-Bi_2_O_3_ phases confined in nanocrystallites embedded in amorphous matrix. Sci. Rep..

[31] Pietrzak T.K., Garbarczyk J.E., Wasiucionek M. (2018). Stabilization of the δ-Bi_2_O_3_-like structure down to room temperature by thermal nanocrystallization of bismuth oxide-based glasses. Solid State Ion..

[32] Nowagiel M., Lafon O., Płociński T., Trébosc J., Turczyński J., Zajkowska-Pietrzak W., Garbarczyk J.E., Wasiucionek M., Pietrzak T.K. (2026). Towards a deeper insight into the stabilisation mechanism of δ-Bi_2_O_3_-like phase to low temperature by confinement of nanograins in glassy matrix. Ceram. Int..

[33] Drozd-Rzoska A. (2005). Pressure dependence of the glass temperature in supercooled liquids. Phys. Rev. E.

[34] Mierzwa M., Paluch M., Rzoska S.J., Zioło J. (2008). The liquid−glass and liquid−liquid Transitions of TPP at elevated pressure. J. Phys. Chem. B.

[35] Drozd-Rzoska A., Rzoska S.J., Starzonek S. (2023). New scaling paradigm for dynamics in glass-forming systems. Prog. Mater. Sci..

[36] Smedskjaer M.M., Bauchy M., Mauro J.C., Rzoska S.J., Boćkowski M. (2015). Unique effects of thermal and pressure histories on glass hardness: Structural and topological origin. J. Chem. Phys..

[37] Svenson M.N., Guerette M., Huang L., Loennroth N., Mauro J.C., Rzoska S.J., Bockowski M., Smedskjaer M. (2016). Universal behavior of changes in elastic moduli of hot compressed oxide glass. Chem. Phys. Lett..

[38] Mascaraque M., Bauchy M., Fierro J.L.G., Rzoska S.J., Boćkowski M., Smedskjaer M.M. (2017). Dissolution kinetics of hot compressed oxide glasses. J. Phys. Chem. B.

[39] Kapoor S., Guo X., Youngman R.E., Hogue C.L., Mauro J.C., Rzoska S.J., Bockowski M., Jensen L.R., Smedskjaer M.M. (2017). Network glasses under pressure: Permanent densification in modifier-free Al_2_O_3_-B_2_O_3_-P_2_O_5_-SiO_2_ systems. Phys. Rev. Appl..

[40] Januchta K., Youngman R.E., Goel A., Bauchy M., Logunov S.L., Rzoska S.J., Bockowski M., Jensen L.R., Smedskjaer M.M. (2017). Discovery of ultra-crack-resistant oxide glasses with adaptive networks. ACS Chem. Mater..

[41] Baranowski P., Starzonek S., Drozd-Rzoska A., Rzoska S.J., Bockowski M., Keblinski P., Pietrzak T.K., Garbarczyk J.E. (2019). Multifold pressure-induced increase of electric conductivity in LiFe_0.75_V_0.10_PO_4_ glass. Sci. Rep..

[42] Starzonek S., Szpakiewicz-Szatan A., Rzoska S.J., Drozd-Rzoska A., Boćkowski M., Pietrzak T.K., Garbarczyk J.E. (2023). Pressure-driven relaxation processes in nanocomposite ionic glass LiFe_0.75_V_0.10_PO_4_. J. Non-Cryst. Solids.

[43] Szpakiewicz-Szatan A., Starzonek S., Pietrzak T.K., Garbarczyk J.E., Rzoska S.J., Boćkowski M. (2022). Novel high-pressure nanocomposites for cathode materials in sodium batteries. Nanomaterials.

[44] Szpakiewicz-Szatan A., Starzonek S., Garbarczyk J.E., Pietrzak T.K., Boćkowski M., Rzoska S.J. (2024). AC Electric Conductivity of High Pressure and High Temperature Formed NaFePO_4_ Glassy Nanocomposite. Nanomaterials.

[45] Szpakiewicz-Szatan A., Pietrzak T.K., Boćkowski M., Rzoska S.J., Garbarczyk J.E., Starzonek S. Title of High pressure treatment of sodium olivine-like glass: Possible future cathode material. Proceedings of the the 3rd Polish-Slovenian International Seminar on Soft Matter.

[46] Yashima M., Ishimura D. (2003). Crystal structure and disorder of the fast oxide-ion conductor cubic Bi_2_O_3_. Chem. Phys. Lett..

[47] Blower S.K., Greaves C. (1988). The structure of β-Bi_2_O_3_ from powder neutron diffraction data. Acta Cryst..

[48] Aurivillius B. (1949). Mixed Bismuth Oxides with Layer lattices I. The structure type of CaNb_2_Bi_2_O_9_. Ark. Kemi.

[49] Fei Y.T., Fan S.J., Sun R.Y., Xu J.Y., Ishii M. (2000). Crystallizing behavior of Bi_2_O_3_-SiO_2_ system. J. Mater. Sci. Lett..

[50] Ivanov S.A., Tellgren R., Rundlof H., Orlov V.G. (2001). Structural Studies of α-Bi_2_O_3_ by Neutron Powder Diffraction. Powder Diffr..

[51] Gunnar M., Liv F., Ballhausen C.J., Ragnarsson U., Rasmussen S.E., Sunde E., Sørensen N.A. (1970). The Crystal Structure of alpha-Bi_2_O_3_. Acta Chem. Scand..

[52] Kalbarczyk A.S., Kalabinski J., Nowinski J.L., Ślubowska W., Wasiucionek M., Garbarczyk J.E. (2015). Properties of 15Ag_2_O·70V_2_O_5_·15P_2_O_5_ glass prepared by melt quenching, twin rollers and mechanosynthesis method. Solid State Ion..

[53] Pietrzak T.K. (2019). Multi-device software for impedance spectroscopy measurements with stabilization in low and high temperature ranges working under Linux environment. Ionics.

[54] Miranda M.A.R., Sasaki J.M. (2018). The limit of application of the Scherrer equation. Acta Cryst..

[55] Honders A., Broers G.H.J. (1985). Bounded diffusion in solid solution electrode powder compacts. Part I. The interfacial impedance of a solid solution electrode (M_x_SSE) in contact with a m^+^-ion conducting electrolyte. Solid State Ion..

[56] Zurhelle A.F., Stehling W., Waser R., De Souza R.A., Menzel S. (2022). Oxygen diffusion in platinum electrodes: A molecular dynamics study of the role of extended defects. Adv. Mater. Interfaces.

[57] Gateman S.M., Gharbi O., Gomes de Melo H., Ngo K., Turmine M., Vivier V. (2022). On the use of a constant phase element (CPE) in electrochemistry. Curr. Opin. Electrochem..

[58] Opitz A.K., Fleig J. (2010). Investigation of O_2_ reduction on Pt/YSZ by means of thin film microelectrodes: The geometry dependence of the electrode impedance. Solid State Ion..

[59] Wallauer J., Balabajew M., Roling B., Endres F., Abbott A., MacFarlane D. (2017). Impedance spectroscopy on electrode|ionic liquid interfaces. Electrodeposition from Ionic Liquids.

[60] Wang Y., Nowick A.S. (1980). The “grain-boundary effect” in doped ceria solid electrolytes. J. Solid State Chem..

[61] Wang D., Park D., Griffith J., Nowick A. (1981). Oxygen-ion conductivity and defect interactions in yttria-doped ceria. Solid State Ion..

[62] Etsell T.H., Flengas S.N. (1970). Electrical properties of solid oxide electrolytes. Chem. Rev..

[63] Shannon R.D., Prewitt C.T. (1969). Effective ionic radii in oxides and fluorides. Acta Cryst..

[64] Zhang J., Lenser C., Menzler N.H., Guillon O. (2020). Comparison of solid oxide fuel cell (SOFC) electrolyte materials for operation at 500 °C. Solid State Ion..

[65] Steele B.C. (1992). Oxygen ion conductors and their technological applications. Mater. Sci. Eng. B.

[66] Zhu B. (2009). Solid oxide fuel cell (SOFC) technical challenges and solutions from nano-aspects. Int. J. Energy Res..

[67] Yeh T.H., Hsu W.C., Chou C.C. (2005). Mechanical and electrical properties of ZrO_2_ (3Y) doped with RENbO_4_ (RE = Yb, Er, Y, Dy, YNd, Sm, Nd). J. Phys. IV.

[68] Jung D.W., Duncan K.L., Wachsman E.D. (2010). Effect of total dopant concentration and dopant ratio on conductivity of (DyO_1.5_)_x_ − (WO_3_)_y_ − (BiO_1.5_)_1−x−y_. Acta Mater..

[69] Xia C. (2002). Microstructures, conductivities, and electrochemical properties of Ce_0.9_Gd_0.1_O_2_ and GDC–Ni anodes for low-temperature SOFCs. Solid State Ion..

[70] Kumar A., Jaiswal A., Sanbui M., Omar S. (2017). Oxygen-ion conduction in scandia-stabilized zirconia-ceria solid electrolyte (xSc_2_O_3_ − 1CeO_2_ − (99 − x)ZrO_2_, 5 ≤ x ≤ 11). J. Am. Ceram. Soc..

[71] Ishihara T., Matsuda H., Takita Y. (1994). Doped LaGaO_3_ perovskite type oxide as a new oxide ionic conductor. J. Am. Chem. Soc..

[72] Gattow G., Schröder H. (1962). Über Wismutoxide. III. Die Kristallstruktur der Hochtemperaturmodifikation von Wismut(III)-oxid (δ-Bi_2_O_3_). Z. Anorg. Allg. Chem..

[73] Takahashi T., Iwahara H., Nagai Y. (1972). High oxide ion conduction in sintered Bi_2_O_3_ containing SrO, CaO or La_2_O_3_. J. Appl. Electrochem..

[74] Takahashi T., Iwahara H., Arao T. (1975). High oxide ion conduction in sintered oxides of the system Bi_2_O_3_-Y_2_O_3_. J. Appl. Electrochem..

[75] Takahashi T., Iwahara H. (1978). Oxide ion conductors based on bismuthsesquioxide. Mater. Res. Bull..

[76] Hochrein O., Zahn D. (2009). Atomic mechanisms of superionic conductivity in fluorite. Solid State Ion..

[77] Kuang X., Payne J.L., Johnson M.R., Evans R.I. (2012). Remarkably high oxide ion conductivity at low temperature in an ordered fluorite-type superstructure. Angew. Chem. Int. Ed. Engl..

[78] Cazorla C., Errandonea D. (2016). Giant mechanocaloric effects in fluorite-structured superionic materials. Nano Lett..

[79] Mahan G.D., Mahan G.D., Roth W.L. (1976). Theoretical Issues in Superionic Conductors. Superionic Conductors.

[80] Derrington C.E., O’keeffe M. (1973). Anion conductivity and disorder in lead fluoride. Nat. Phys. Sci..

[81] Voronin B.M., Volkov S.V. (2001). Ionic conductivity of fluorite type crystals CaF_2_, SrF_2_, BaF_2_, and SrCl_2_ at high temperatures. J. Phys. Chem. Solids.

[82] Ravi B.G., Baskaran N., Ramasamy S. (1997). Effect of anion disorder on the ionic conductivity of CaF_2_ single crystals. Mater. Chem. Phys..

[83] Funke K., Banhatti R.D. (2007). Ionic transport and localized ionic motion in Na-β″-alumina, Na_1.70_Li_0.32_A_l10.66_O_17_. J. Mater. Sci..

[84] Park S.M., Hellstrom E.E. (1994). Ionic conductivity of Na, K and Ag β″-alumina-ZrO_2_ composites. J. Mater. Sci..

[85] Farrington G. (1982). Divalent beta″-aluminas: High conductivity solid electrolytes for divalent cations. Solid State Ion..

[86] Carr V.M., Chadwick A.V., Saghafian R. (1978). The electrical conductivity of PbF_2_ and SrCl_2_ crystals at high temperatures. J. Phys..

[87] Chadwick A. (1983). High-temperature transport in fluorites. Solid State Ion..

[88] Castiglione M.J., Wilson M., Madden P.A., Grey C.P. (2001). Ion mobility in α-PbF_2_: A computer simulation study. J. Phys. Condens. Matter.

[89] Balluffi R.W. (1978). Vacancy defect mobilities and binding energies obtained from annealing studies. J. Nucl. Mater..

